# Sternocleidomastoid flap for pedicled reconstruction in head & neck surgery- revisiting the anatomy and technique

**DOI:** 10.1186/s12957-021-02470-5

**Published:** 2021-12-20

**Authors:** Apurva Srivastava, Tarun Kumar, Shashi Kant Pandey, Ram Chandra Shukla, Esha Pai, Manoj Pandey

**Affiliations:** 1 Department of Vascular Surgery, Sir Gangaram Hospital, New Delhi, India; 2 Department of Surgical Oncology, Institute of Medical Sciences, Banaras Hindu University, Varanasi, India; 3 Department of Anatomy, Institute of Medical Sciences, Banaras Hindu University, Varanasi, India; 4 Department of Radiology, Institute of Medical Sciences, Banaras Hindu University, Varanasi, India; 5 Department of Surgical Oncology, Heritage Hospital, Varanasi, India

**Keywords:** Sternocleidomastoid, flap, Head and Neck Cancer, Reconstruction, Cadaver, Angiography, Superior Thyroid Artery

## Abstract

**Background:**

Previous studies on sternocleidomastoid flaps, have defined the importance of preserving sternocleidomastoid (SCM) branch of superior thyroid artery (STA). This theory drew criticism, as this muscle is known to be a type II muscle, i.e., the muscle has one dominant pedicle (branches from the occipital artery at the superior pole) and smaller vascular pedicles entering the belly of muscle (branches from STA and thyrocervical trunk) at the middle and lower pole respectively. It was unlikely for the SCM branch of STA to supply the upper and lower thirds of the muscle. We undertook a cadaveric angiographic study to investigate distribution of STA supply to SCM muscle.

**Methods:**

It is a cross-sectional descriptive study on 10 cadaveric SCM muscles along with ipsilateral STA which were evaluated with angiography using diatrizoate (urograffin) dye. Radiographic films were interpreted looking at the opacification of the muscle. Results were analyzed using frequency distribution and percentage.

**Results:**

Out of ten specimens, near complete opacification was observed in eight SCM muscle specimens. While one showed poor uptake in the lower third of the muscle, the other showed poor uptake in the upper third segment of muscle.

**Conclusion:**

Based on the above findings we suggest to further investigate sternocleidomastoid muscle as a type III flap, as the STA branch also supplies the whole muscle along with previously described pedicle from occipital artery. However, this needs to be further corroborated intra-operatively using scanning laser doppler. This also explains better survival rates of superior thyroid artery based sternomastoid flaps.

## Introduction

Guidelines recommend microsurgical free flap reconstruction as a primary reconstructive option for most defects of head and neck surgery [1, 2]. The use of free flaps is based on the tenet of importing large volumes of healthy tissue from sites distant from radiotherapy and surgical fields. Free radial forearm and free anterolateral thigh flap are the most commonly used microvascular flaps for the soft tissue cover [1]. The only downside being the donor site morbidity [1]. However, this does not reduce the importance of learning the techniques of pedicled flaps for a head and neck surgeon. When the situation demands or when means and expertise are not available, the surgeons have to fall back on pedicled flaps. One such flap is the pedicled sternocleidomastoid (SCM) myocutaneous flap.

Conventionally, the SCM flap has been described as a type II flap by Mathes and Nahai [3] (Table 1), i.e. the muscle has one dominant pedicle (branches from the occipital artery at the superior pole) and smaller vascular pedicles entering the belly of muscle (branches from superior thyroid artery (STA) and thyrocervical trunk) at the middle and lower pole, respectively.

**Table 1:** Patterns of muscle circulation as described by Mathes & Nahai

	Type of vascular pedicle	Example
Type I	One Vascular Pedicle	Gastrocnemius, Rectus femoris, Tensor fascia lata
Type II	Dominant vascular pedicle plus minor pedicles	Temporalis, Trapezius, Sternocleidomastoid, Gracilis
Type III	Two Dominant Pedicles	Gluteus Maximus, Rectus Abdominis, Serratus Anterior, Semimembranosus
Type IV	Segmental vascular pedicles	Sartorius, Tibialis anterior, Extensor hallucis longus, Flexor Digitorum longus, Extensor digitorum longus, Flexor hallucis longus
Type V	One dominant pedicle and secondary segmental pedicles	Pectoralis major, Latissimus dorsi

The SCM flap is used to reconstruct defects of cheek, floor of mouth [4–6], mandible [7] and defects after parotidectomy [8–10]. It is also used to reconstruct oropharyngeal and hypopharyngeal wall [11–14], laryngotracheal complex [15–18], close pharyngocutaneous and cervical oesophagus fistula [19–22].

This flap is usually not the preferred choice of most surgeons because of it’s proximity to internal jugular lymph nodes, risk of damage to blood supply during neck dissection and ill-effects of radiotherapy on flap viability [20, 23]. Total or partial flap loss has been reported in 10-30% of the cases [24]. However, flap loss may be minimized by modifying the technique to preserve the branch from superior thyroid artery (STA), as shown in our own series of 32 patients, resulting in flap loss of 6.25% [25]. The branch from STA is preserved, firstly by identifying the branch arising from the STA and then dissecting distal to its take off from STA, close to the thyroid gland and if required ligating the STA on the thyroid gland in order to provide the desired length to the flap.

As classically described, if the SCM muscle is a Type II muscle (i.e., having one major and two segmental minor pedicles) then technically, preserving the STA branch should not improve flap results. This was the criticism our previous series received. Hence, we decided to test the hypothesis that the STA pedicle alone would offer adequate perfusion of the muscle, in the absence of perfusion by the posterior occipital artery.

With the above background, we decided to re-visit the arterial anatomy of this flap with the help of cadaveric angiography to test the hypothesis.

Cadaveric angiography is a known technique to study the arterial anatomy of muscles and has been used previously by various other authors [26, 27]. We chose to test our hypothesis using the cadaveric angiography because in a cadaver it was possible to study only the perfusion of the SCM branch of STA. This would be not be possible by conventional angiography as the perfusion from other pedicles (posterior occipital and branches from transverse cervical artery) would confound the results.

## Methods

**Fig. 1:**
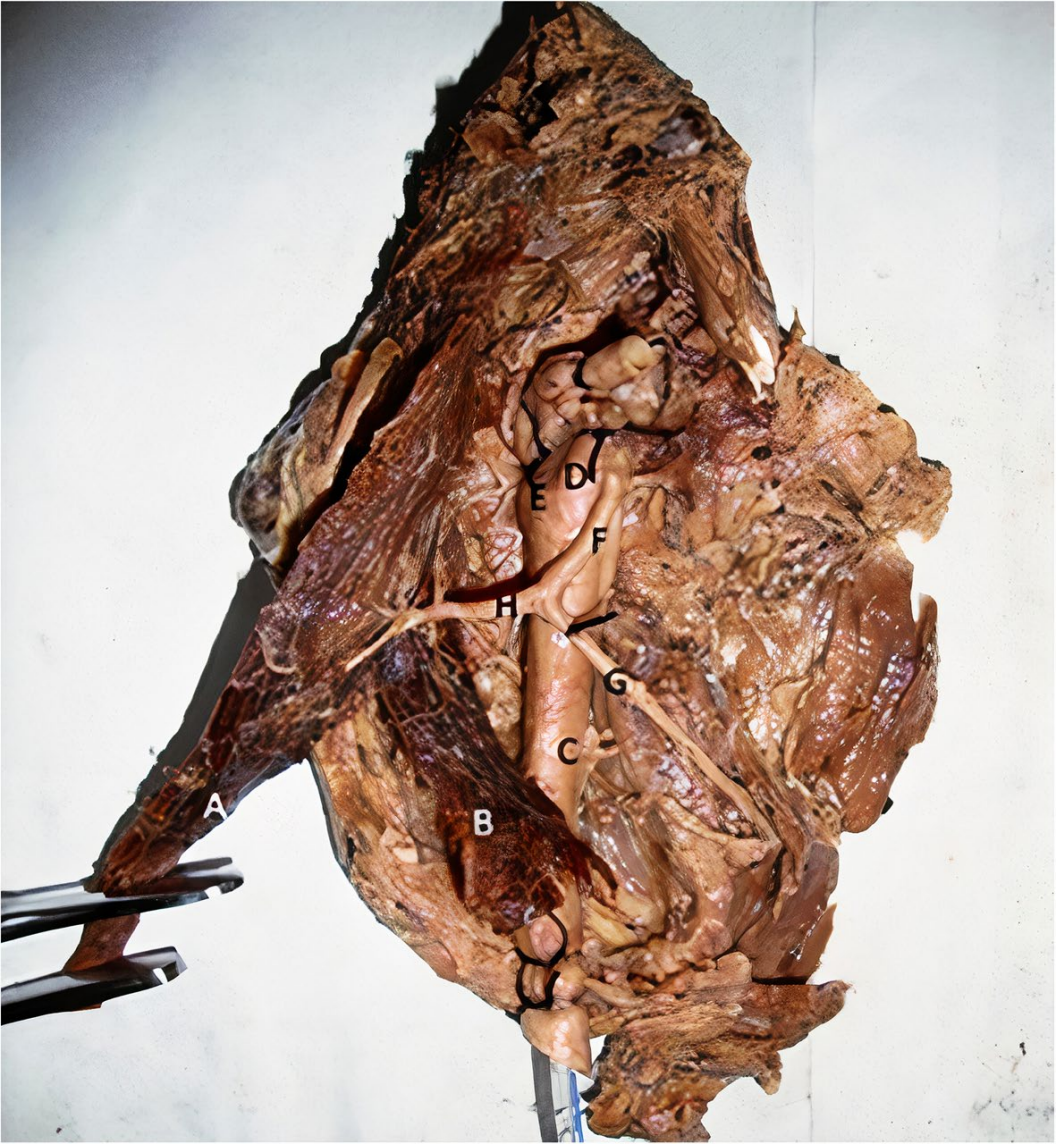
Specimen of SCM muscle with its blood supply. Infant feeding tube can be seen in common carotid artery. Internal carotid artery and external carotid artery distal to STA is ligated. STA distal to SCM branch is also ligated. (**A**: Clavicular head of sternocleidomastoid, **B**: Sternal head of sternocleidomastoid, **C**: Common carotid artery, **D**: External carotid artery, **E**: Internal carotid artery, **F**: Superior thyroid artery, **G**: Thyroid branch of superior thyroid artery, **H**: Sternomastoid branch of superior thyroid artery), **I**: Fibrofatty tissue of the neck

The study was conducted at the Department of Anatomy, Institute of Medical Sciences, Banaras Hindu University, Varanasi, India. This was a cross-sectional descriptive study where angiographic examination of the SCM muscle was performed on dissected specimens from cadavers to evaluate its blood supply. All the cadavers used were fresh i.e., with 5 hours of death. The neck was dissected immediately and the sternomastoid muscle with the carotid artery were taken out as a unit and the angiographic study was performed ex-vivo. 10 specimens were dissected out from 5 cadavers, i.e., both right sided and left sided specimens. In the specimen, we included (Fig. 1):

i. The common carotid artery (CCA) and its bifurcation into the external and internal carotid arteries (ECA & ICA).
ii. The STA, along with its origin from the external carotid artery.
iii. The SCM of the same side, keeping its pedicles from STA intact.

After placing an infant feeding tube into the common carotid artery, lumen of the all the vessels was flushed with normal saline until no more clots were seen. After removing the clots vessels were ligated at three different sites:

i. At the proximal end of the common carotid artery.
ii. At the distal end, both on the external and internal carotid arteries.
iii. At the distal end of the STA, after the branch to SCM.

Angiography was done immediately after the dissection was completed and the specimen was prepared as described above.

A 20 milliliters (ml) suitable contrast material, 76% diatrizoate (Urograffin) was injected as a bolus push, using a 20 ml syringe through the infant feeding tube from the proximal end. After 5 minutes, keeping it in anatomical position i.e., position of the muscle with its blood supply as it is in the human body, a radiograph of the specimen was obtained. Radiograph was taken using x- rays at 50 kV and 20 mAs. Only outcome measure studied was the pattern of muscle perfusion.

Radiographic films were interpreted looking at the opacification of the muscle. A radiolucent hue was considered as perfusion of the muscle segment.

Atherosclerosis if present usually affects the bifurcation of wider arteries i.e., CCA bifurcating into ECA and ICA. The branching of STA from ECA is usually not the site of atherosclerotic plaques [28].

Data was analyzed using frequency distribution and percentage using Microsoft Excel. Institutional ethics committee approval was obtained before starting the study.

## Results

**Fig. 2:**
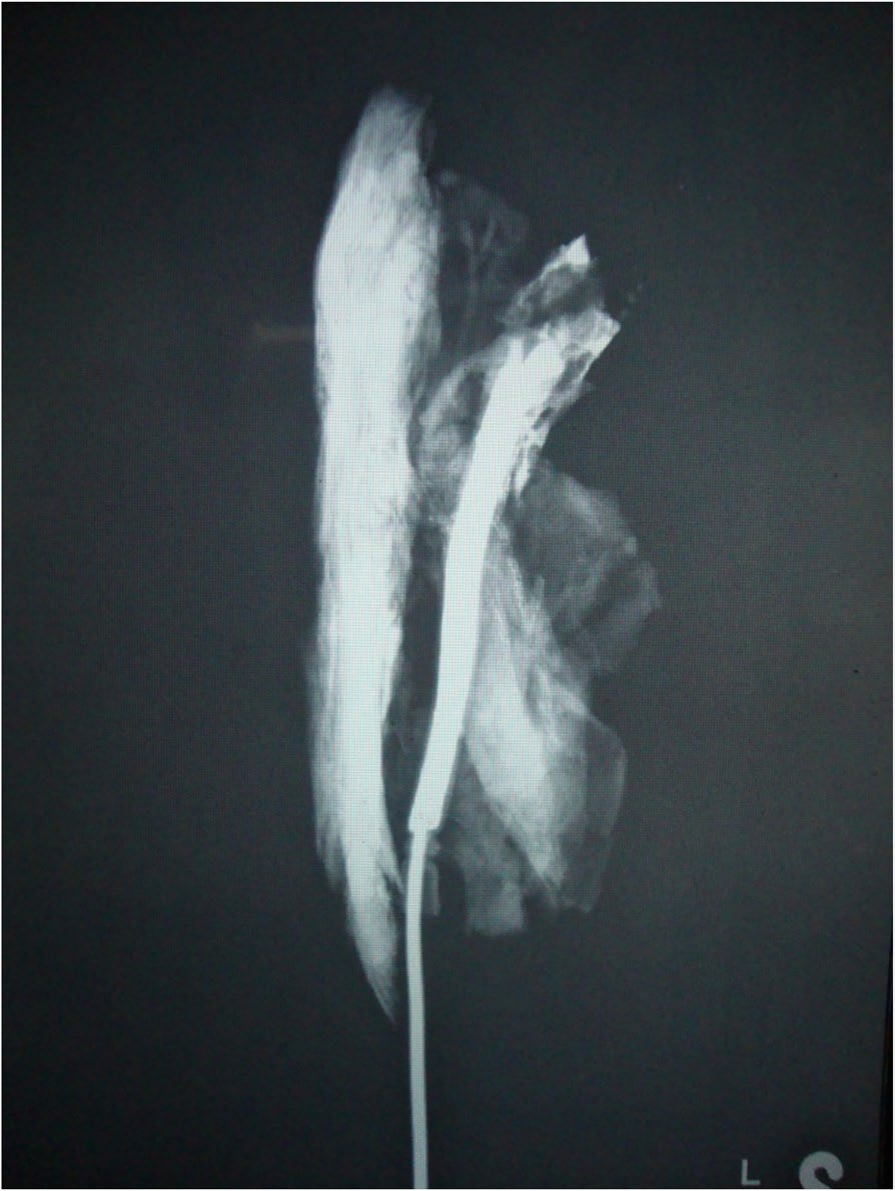
Angiographic film showing complete opacification of the muscle

**Fig. 3:**
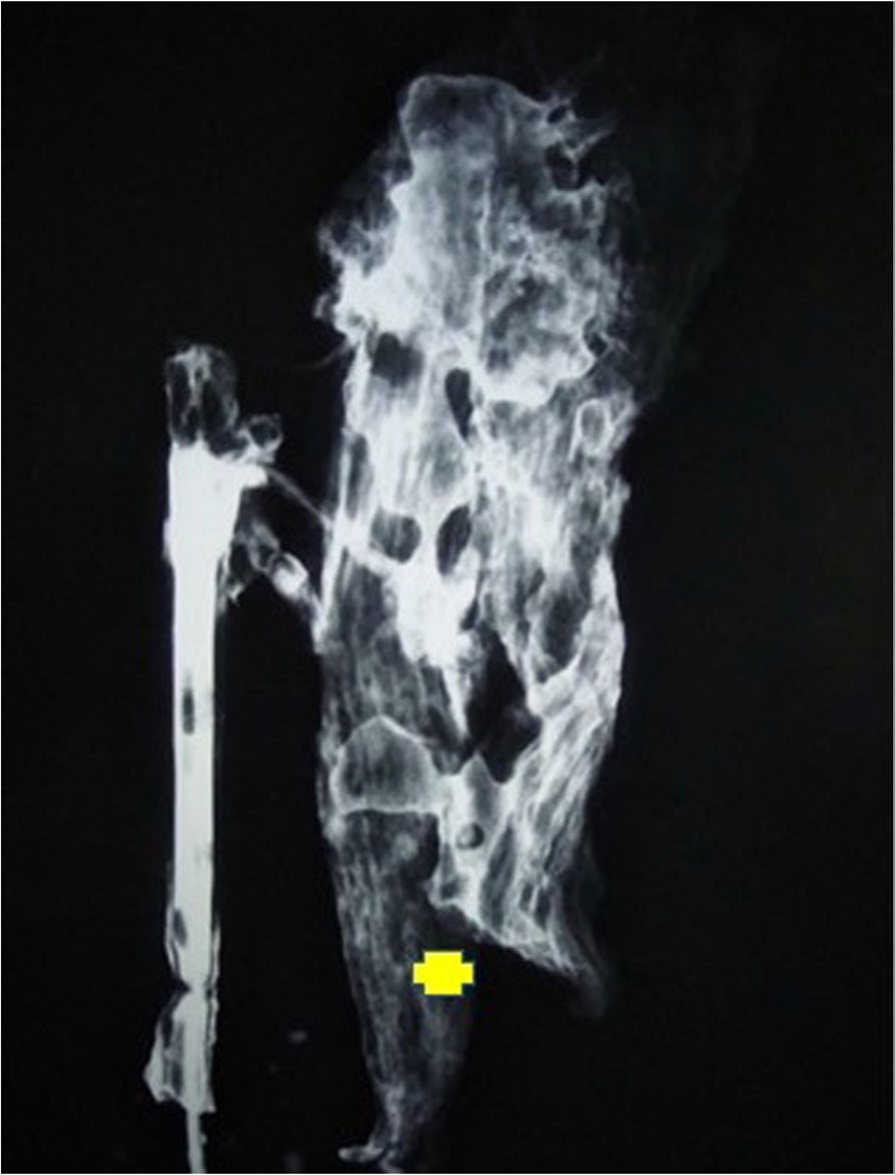
Angiographic film showing un-opacified lower third. Yellow cross indicates the un-opacified region

**Fig. 4:**
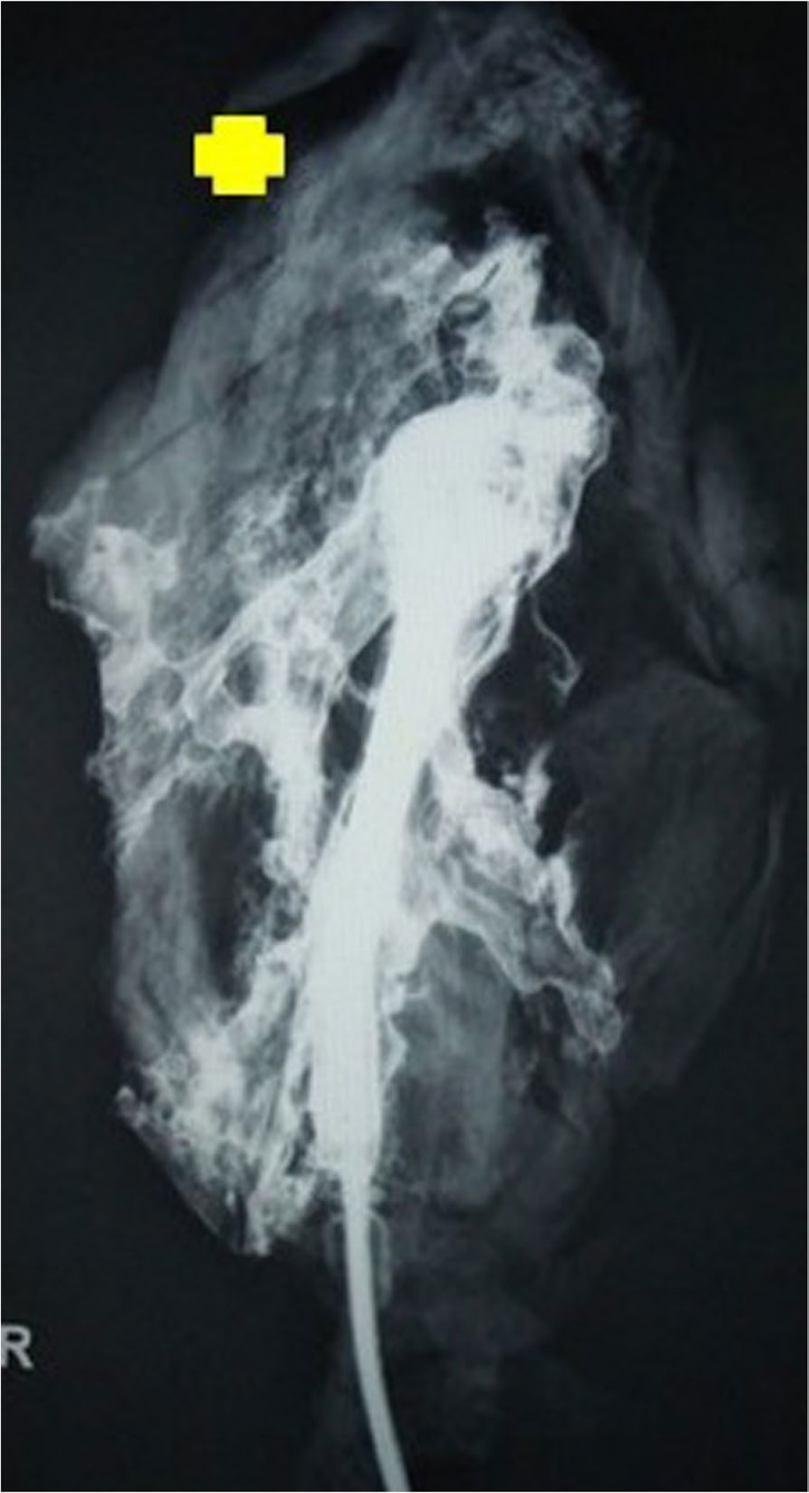
Angiographic film showing un-opacified upper third. Yellow cross indicates the un-opacified region

Out of ten specimens, we saw near complete opacification of eight SCM muscle specimens (Fig. 2). One specimen showed poor uptake in the lower third of the muscle, and another showed poor uptake in the upper third segment of muscle (Figs. 3 and 4)

All eight specimens wherein the whole muscle was opacified, showed uniform uptake in all the three segments of the muscle.

## Discussion

Based on the above findings, it seems that the SCM perfusion resembles that of a type III muscle, than of type II, as described previously in literature [3]. A type III flap has two dominant pedicles. According to the findings in this study, the STA is the second dominant pedicle supplying the SCM flap, other than its already established supply from the occipital artery (which serves as its first dominant pedicle). Our findings might imply, that STA, not only augments the blood supply of the muscle (via occipital artery) but, is also capable of solely supplying the whole muscle in 80% of cases. STA supplied two-thirds of the muscle, including the lower third of the muscle, in 90% cases. In our previous series, we reported a flap loss of 6.25 % [25], by preserving the SCM branch of STA, as opposed to the historical 10-30%, reported in the literature [24]. These clinical results are reinforced by our present angiographic study. In situations, where more length of the flap is required, the STA was ligated distal to the SCM branch [25]. Tiwari et al have described in their series of 18 successful cases of SCM flaps, of which 3 flaps were done for temporal bone resection defects [22]. In these 3, they detached the muscle from the temporal bone, and none of the muscular branches of occipital artery were preserved. The flap survived solely on the SCM branch from STA, as well as collateral circulation from the branches of occipital and posterior auricular arteries supplying the overlying skin. Wei et al., in their series of 65 patients, emphasized on preserving the SCM branch of STA. They reported partial flap loss of 8 % and did not report any total flap loss [29]. Similarly, Khazaeni in their lone case [30], reported sternocleidomastoid myocutaneous flap, for a full thickness cheek defect solely based on the SCM branch of STA, as the occipital branch was ligated during the lymph-node dissection in the same case. Jones et al. [24] in their systematic review have described decrease in the complications of the SCM flap from about 60 % [12] in 1980s to present rate of about 2% (2016) [31]. In their review, they attributed lower complication rates to: 1) suturing skin paddle to underlying muscle so that the perforators can be safeguarded from shearing, while handling the flap 2) to check the skin refill before suturing the flap to the defect 3) preservation of SCM branch of superior thyroid artery.

Hu et al dissected 50 cadaveric muscle specimens (without angiography), described a branch of STA, that runs down to the clavicle and supplies perforators to perfuse the lower third segment of the muscle. They also stressed on the importance of STA in supplying the lower third of the muscle [32].

The relatively higher loss of the flap in various series may be due to poor underlying perforators to the overlying skin in which case, there is only a partial loss of the skin paddle while the underlying muscle remains healthy. This has been discussed in detail by Jabaley et al. where they studied the blood supply of sternocleidomastoid in 3 ways: cadaveric dissection, neck dissections and fluorescein dye testing, after the flaps were raised. They concluded that due to paucity of perforators from the muscle to skin or very narrow caliber capillaries traversing from muscle to overlying skin, there is a greater chance of skin-paddle loss that would eventually heal by re-epithelization with healthy underlying muscle [33]. Another proposed reason for higher flap loss is disrupted venous drainage. As most of the discussions revolve around arterial anatomy of the muscle, venous drainage is often overlooked. SCM is drained by accompanying venous tributaries with each vascular pedicle and additionally by small tributaries to external and anterior jugular vein [34]. Every effort should be made to preserve maximum venous tributaries to prevent congestion of the flap.

The limitation of our study is a relatively small sample size, that may obviate anatomic variations in the blood supply and this requires larger numbers to be studied. Another drawback is the unaccounted entity of “feeding-vessel” spasm, a phenomenon which may get aggravated by flap-handling [35–37]. In real time surgery, there is always a chance of feeding vessels or perforators ‘going into spasm’ despite rich supply through intramuscular communications. As with all cadaveric studies, it was not possible to study this and it may very well be a cause of flap failure despite angiographic uptake in the lower third of the muscle.

## Conclusion

Our findings hint towards possibility of type III like distribution of blood vessels in the SCM muscle instead of type II. We suggest to further investigate SCM muscle’s blood supply using larger number of subjects. This should be further corroborated with intra-operative scanning laser doppler after occluding occipital artery.

We also strongly recommend to preserve STA branch to the SCM along with the occipital artery for better perfusion of lower third of the flap, hence minimizing rates of flap loss and flap-related morbidity.

## Data Availability

On Request
